# Antibiotic Prescribing Trends Before and After Implementation of an Audit and Feedback Program in Internal Ward of a Tertiary Hospital in Tehran

**DOI:** 10.22037/ijpr.2019.1100833

**Published:** 2019

**Authors:** Ensieh GolAli, Mohammad Sistanizad, Jamshid Salamzadeh, Mehrdad Haghighi, Mehrdad Solooki

**Affiliations:** a *Department of Clinical Pharmacy, Faculty of Pharmacy, Shahid Beheshti University of Medical Sciences, Tehran, Iran. *; b *Department of Pharmaceutical Care Unit, Emam Hossein Medical and Educational Center, Shahid Beheshti University of Medical Sciences, Tehran, Iran. *; c *Food Safety Research Center, Shahid Beheshti University of Medical Sciences, Tehran, Iran. *; d *Department of Infectious Diseases, Imam Hossein Teaching and Medical Hospital, Shahid Beheshti University of Medical Sciences, Tehran, Iran. *; e *Department of Pulmonary and Critical Care Medicine, Imam Hossein Hospital, Shahid Beheshti University of Medical Sciences, Tehran, Iran.*

**Keywords:** Audit and Feedback Program, Appropriate antibiotic use, Antibiotic stewardship, Consumption rate, Patient outcomes

## Abstract

We implemented a post prescribing review and feedback program to investigate its effect on appropriateness of antimicrobial use and antimicrobial consumption rate. A pre-post interventional study conducted in internal ward of Imam Hossein teaching hospital. For nine months of intervention phase, medical file of all patients who received intravenous antibiotic were reviewed by a clinical pharmacy specialist. Discrepancies from international and local guidelines were discussed with physicians. Outcome measures included appropriateness of antimicrobial usage, length of stay, and broad-spectrum antimicrobial usage rate. A total of 198 antibiotic courses (154 in intervention phase and 44 in pre-intervention phase) were reviewed. One-hundred sixty-seven recommendations in treatment course of 75.3% of patients were made. The most common recommendations were discontinuing antibiotics and changing from intravenous to oral therapy (35% and 22%). The acceptance rate was 80.2%. Rate of discrepancies from guidelines was compared between pre-intervention and two last months of intervention period which showed a significant reduction in antibiotic choosing (47%, *P*-value < 0.001), de-escalation (48%, *P*-value < 0.001), on time changing intravenous to oral therapy (60%, *P*-value < 0.001) and dosing schedule (30%, *P*-value = 0.003). Hospital length of stay showed a significant reduction from 16.1 days to 11.6 days (*P*-value < 0.05) between pre-intervention and post-intervention group. Mortality rate was not different in the patients that intervention in their treatment was accepted *vs.* rejected (*P*-value = 1.00). There was a reduction trend in consumption rate of Carbapenems, Vancomycin, and Ciprofloxacin.

Therefore, prospective audit and feedback program effectively decreased inappropriate treatment and hospital length of stay with no effect on mortality.

## Introduction

Antibiotic stewardship program (ASP) has been defined as “coordinated interventions designed to improve and measure the appropriate use of antibiotic agents by promoting the selection of the optimal drug regimen including dosing, duration of therapy, and route of administration” (1). Prospective audit with intervention and feedback to prescribers can reduce inappropriate antimicrobial use and is recognized as one of the main parts of ASP (2). While in many countries a multidisciplinary ASPs have been implemented in most major hospitals for years, there have been only limited actions such as “formulary restriction” in some hospitals in Iran (3). In past few years many hospitals in Iran started to give attention to importance of applying a program for controlling antimicrobial consumption. Also, parallel to ASPs, finding new regimens for treatment of resistant bacteria were focused by some researchers (4, 5).

“Prospective audit and feedback” has been designed in very different ways using dissimilar approaches based on services involved on ASP (infectious disease specialist, clinical pharmacist, etc.), history of ASP implementation in the hospital, average patients’ length of stay (long-term acute care hospitals *vs.* regular hospitals), resources, etc. (6-12). 

In contrast to “formulary restriction” making a predictable reduction in antimicrobial consumption, “prospective audit and feedback” effects are dependent on applied program and they need to investigate the best approach based on local conditions. Financial constraint and not having a dedicated team, emphasizes the need for finding the simplest effective approach.

Based on our knowledge this is the first trial evaluating effect of implementing “prospective audit and feedback” intervention on antimicrobial use in Iran.

## Experimental

*Setting*

This prospective interventional study was done in Imam Hossein teaching hospital, affiliated to Shahid Beheshti University of Medical Sciences, Tehran, Iran. Based on antimicrobial consumption and diversity of the patients, internal medicine ward with 46 beds and occupancy rate of 96%, including general, nephrology, pulmonology, endocrinology, and hematology sub-specialties was selected for implementing prospective audit and feedback intervention. Patients were visited in teaching rounds every day in this ward by responsible attending ward staphs. Leading infectious causes are pulmonary infections (45%), skin and soft tissue infections, urinary tract infections, and catheter related infections in dialysis patients.”

*Inclusion and exclusion criteria*

In this pre-post interventional study, all patients of the receiving an intravenous antimicrobial were included in the study. The immunocompromised patients (HIV patients, neutropenic patients and those hospitalized for chemotherapy and others with known immunocompromised situations), and the patients without definite diagnosis based on guidelines were excluded.

*Study design*

Pre-interventional phase was done between January to March 2017 for three months. A clinical pharmacy specialist screened the patients based on defined inclusion and exclusion criteria three times weekly. Clinical pharmacist visited all patients included in the study, three times weekly. Data related to diagnosis and treatment of infection including chief complaint, sign, and symptoms suggesting an infection, laboratory data (including WBC count and differential, ESR, CRP, PCT, BUN, SCr, urinary analysis, VBG, CFS analysis, pleural fluid analysis, etc.), imaging results (CXR, sonography report, etc.), results of culture and susceptibility tests and history of recent antibiotic use, receiving immunosuppressing drugs and recent hospital admissions were documented. Any change in treatment (changing antibiotic, dose adjusting, de-escalation, changing route of administration, and discontinuation documented. Ward staphs were not aware of study method and goals in this stage. 

After completing data in pre-interventional phase, based on extracted data (frequencies of infectious disease and detected routine wrong ways of antibiotic use), protocols for treatment of prevalent infections including protocols for diagnosis and treatment of pneumonia (community and hospital acquired), sepsis, and diabetic foot infection were designed by a team including infectious disease and clinical pharmacy specialists and infection control supervisor. These protocols determine the time feasible to start antibiotic treatment, the antibiotic that should be chosen (Fluoroquinolones and Carbapenems eliminated from first line antibiotics whenever it was passible based on hospital policy in order to control resistance of microorganisms to them), the preferred dose and route of administration, how the patient should be follow up, and the right duration of the treatment. All guidelines were approved by drug and therapeutics committee before implementation of prospective audit and feedback protocol.

In interventional phase, between June 2017 and February 2018, clinical pharmacy specialist visited the patients and discussed with prescriber in teaching rounds in case of deviation from pre-designed guidelines. In patients for whom the clinical pharmacist could not decide, before any recommendation to physician in charge, the consultation with infectious disease specialist was done. The Physicians were free to accept the recommendations or not. At the beginning of the audit and feedback program, we planned to discuss necessary modifications with responsible physician. They were available only in five concurrent teaching rounds for two hours in the morning, which made it impossible to attend in all visits. We continued this strategy for 2 months until September 2017. Then we decided to try another strategy and for 3 months, between September 2017 to December 2017, after documenting recommendations in audit forms and putting them in patients file, we discussed them with senior residents and asked them to transfer ASP team opinion to physician in charge. Unfortunately, follow up 48 h after documenting the recommendation revealed that in most cases the recommended modification was not applied and after communication with physician, he/she denied recommendation transfer in most cases. Introducing the plan and its aim to residents in teaching classes did not make any difference so we changed our approach to primary way after November 2017. 

*Outcome measures*

The primary objective of the current study was evaluating appropriateness of antimicrobial consumption before and after implementing prospective audit and feedback intervention, based on seven categories defined in Table 1. As secondary outcome, length of hospital stay, mortality rate, and defined daily dose of antimicrobials per 100 bed days were evaluated in two phases of the study.

*Statistical Analyses*

Categorical variables were analyzed by χ^2 ^or Fisher’s exact tests and Pearson Chi-Square test. Continuous data are presented as the mean ± standard deviation and were analyzed by Student’s *t*-test or Mann–Whitney test. Two-tailed *P*-value of <0.05 was considered statistically significant. All of the collected data were analyzed using IBM SPSS Statistics for Windows v.21.0 (IBM Corp., Armonk, NY).

## Results

During pre-intervention phase the data related to 44 patients were analyzed. The most commonly used intravenous (IV) and oral (PO) antimicrobials were Levofloxacin (IV and PO), Ciprofloxacin (IV and PO), Ceftriaxone (IV), Meropenem (IV), Clindamycin (IV and PO), and Vancomycin (IV). 

In post intervention phase which lasts 9 months, 154 patients were included that 116 (75.3%) of them had at least one discrepancy based on the approved guideline. In total 167 (1.08 per patient) recommendations were discussed with responsible physician and 134 (80.2%) of them were accepted. Among 167 recommendations, IV to oral conversion had highest rate (22.2%) and dosing schedule had highest rate of acceptance (100%). Number of provided recommendations and their acceptance rates are summarized in Table 2. Demographics characteristics of patients are reported in Table 3.

For determining effect of audit and feedback intervention on physicians’ routine practice in prescribing antimicrobials and minimizing confounding factors specially differences in pattern of infections, we compared rate of discrepancies from guidelines in same months before and after intervention. Therefore, the data related to January and February 2018 were compared to the data in the same months in 2017 (pre intervention phase). Our study revealed significant decrease in discrepancies in choosing antibiotics, dosing schedule, de-escalation, and IV to PO conversion after intervention but change in indication and sending relevant cultures did not show any statistically significant difference. In addition, we were not able to evaluate effect of intervention on duration of treatment because most of the patients were discharged form hospital with an oral antibiotic. These data are summarized in Table 4.

Hospital length of stay showed a significant reduction from 16.1 days to 11.6 days (*P*-value < 0.05) comparing pre- and post-intervention periods. The mortality rate was not different in the patients whose physicians accepted our recommendation(s) compared to those whose physicians rejected our recommendation(s) in their treatment. (*P*-value = 1.00). During the intervention phase, in the patients, for whom de-escalation or discontinuation of antibiotic(s) was recommended and accepted by their physicians, no exacerbation of infection or need for new antibiotic(s) was seen. 

Comparing consumption of common antibiotics, including Carbapenems, Vancomycin, Ciprofloxacin and Levofloxacin using DDD/100 bed days revealed a reducing trend which was not significant. There was a significant reduction in rate of changes in consumption rate of Carbapenems (+5.34 DDD/100 patient bed days (PBD) to -1.7 DDD/100 PBD, *P*-value = 0.003, 95% CI, 2.7-11.3). 

## Discussion

Results of our study revealed that post prescription review of intravenous antibiotic orders, three times weekly by a clinical pharmacy specialist in internal ward significantly reduced discrepancy from guidelines (Table 4) and led to more appropriate treatment without any detrimental effect on treatment outcomes. In addition, this intervention reduced hospital stay significantly with no effect on mortality rate. One explanation for decreasing hospital stay is that physicians generally discharge patients on oral medications but not on treatment with IV antibiotics. As one of our major findings was significant increase in rate of IV to oral conversion, this could be one reason for decreased hospital length of stay in intervention phase of our study. These data are in accordance with other studies, which reveal the effect of IV to Po conversion on reduction of hospital stay (13-15). 

The physicians in this study accepted 81% of our recommendations, which was more than what we expected at the beginning of the study. The effect of activities by antimicrobial stewardship committee in last six years should not be underestimated in that. These activities focused mostly on “formulary restriction”, “education”, and “antimicrobial order forms” and not on prospective audit and feedback strategy. 

**Table 1:** Definition of categories of deviations from guideline in a treatment course

**Category**	**Definition**
1: Indication	Antibiotic was started but clinical, imaging and laboratory data are not convincing for an infection to be the culprit
2: Culture	Specimen(s) was/were not send from susceptible sources of infection for culture and sensitivity test
3: Antibiotic choosing	Infection is susceptible but selection of antibiotic was not appropriate for probable infection
4: Dosing schedule	Antibiotic dose was not appropriate based on patient situation including renal and liver function and type of possible infection such as meningitis
5: De-escalation	De-escalation was not done based on culture results or clinical recovery
6: IV to oral conversion	Changing IV form of antibiotic to PO delayed in a case of normo-thermia for more than 48 h
7: Duration	Antibiotic(s) was/were continued for duration longer than recommended by guideline

**Table 2:** Frequency of recommendations for correcting deviation from guideline and their acceptance rate during 9 month of intervention

**Category of recommendation Portion of total recommendations, N o. (%)**	**Acceptance rate**
1: Indication 24 (14.4%)	66.7%
2: Culture 27 (16.2%)	88.9%
3: Antibiotic choosing 5 (3.0%)	60.0%
4: Dosing schedule 20 (12.0%)	100.0%
5: De-escalation 23 (13.8%)	78.3%
6: IV to oral conversion 37 (22.2%)	81.0%
7: Duration 11 (6.6%)	72.7%
8: Others* 20 (12.0%)	75.0%
Total 167	80.2%

*Asking for more laboratory investigation like checking PCT for discontinuing antibiotics and SCr for dose adjusting.

**Table 3:** Demographics characteristics of patients in pre-intervention and post intervention phases of the study in January and February of 2017 and 2018

**Parameters**		**Pre-intervention**	**Post intervention (39 **	***P*** **-value**
		**(44 patients), No. (%)**	**patients), No. (%)**	
Sex	Male Female	27 17	19 20	
	Average	62.7	64.6	0.75
Age	Range	19-89	25-90	
	Std. deviation	17.3	17.3	
	Metabolic disorder	9 (20.4%)	8 (20.5%)	0.99
	Kidney disease	6 (13.6%)	10 (25.6%)	0.16
	Pulmonary disease	7 (15.9%)	10 (25.6%)	0.27
	Coronary artery disease	8 (18.2%)	6 (15.4%)	0.73
Underlying disease	Neurologic disorders Solid tumor	3 (6.8%) 4 (9%)	2 (5.1%) 1 (2.5%)	1.00 0.36
	Hematologic disorders	1 (2.2%)	0 (0%)	1.00
	Gastrointestinal	1 (2.2%)	0 (0%)	1.00
	Rheumatologic disease	2 (4.5%)	1 (2.5%)	1.00
	None	3 (6.8%)	1 (2.5%)	0.62
	Respiratory infections	19 (43.2)	20 (51.2%)	0.46
	Endocarditis/Bacteremia	5 (11.4%)	5 (12.8%)	0.84
	Urinary tract infection	6 (13.6%)	8 (20.5%)	0.4
Type of infection	Skin and soft tissue	10 (22.7%)	4 (10.25%)	0.13
	Intra-abdominal	1 2.2%)	0	0.53
	Osteomyelitis	2 (4.5%)	0	0.49
	No diagnosis	1 (2.2%)	2 (5.1%)	0.59

**Table 4:** Rate of discrepancies from guideline between pre-intervention and post intervention phases of the study in January and February of 2017 and 2018 as a surrogate of the effect of audit and feedback intervention on physicians’ routine practice in prescribing antimicrobials

**Category of discrepancy**	**Pre-intervention (44 patients), No. (%)**	**Post intervention (39 patients), No. (%)**	***P*** **-value**
1: Indication	5 (11.36%)	5 (12.82%)	1.00
2: Culture	18 (40.90%)	10 (25.64%)	0.168
3: Antibiotic choosing	24 (54.54%)	3 (7.69%)	< 0.001
4: Dosing schedule	19 (43.18%)	5 (12.82%)	0.003
5: De-escalation	30 (68.18%)	8 (20.51%)	< 0.001
6: Conversion to oral regimen	33 (75%)	6 (15.38%)	< 0.001
7: Duration*	-	-	-

*As most of patients discharged from hospital with an oral antibiotic, duration of treatments could not be evaluated.

Reduction in discrepancies compared to pre interventional phase of the study was more prominent in “on time intravenous to oral conversion” (60%). Change in route of administration is called “low-hanging fruit” in implementing ASP as it is the most obtainable target and has demonstrated significant financial savings (16). In the opposite of IV to oral conversion, our study could not decrease discrepancies in discontinuation of antibiotic in the patients having not enough evidence of infection. 

The current study found that DDD/100 bed day did not reduced significantly. This finding is consistent with that of Manuel in 2010 and Elligsen in 2015 which showed that intervention had no significant impact on the overall antibiotic consumption (17, 18). However, the findings of the studies by Newland in 2012 and Carins in 2013 revealed 18% and 10% reduction with implementing prospective audit and feedback strategy (6, 19). This differs from the findings presented here. Also overall number of DDD/100 bed day did not show any significant reduction in our study, but there was a reduction in trend of Vancomycin and Carbapenems usage rate. About Levofloxacin, although consumption rate did not show any change but average number of DDD per each patient decreased from 5.6 ± 1.9 DDD to 3.7 ± 1 DDD (*P*-value = 0.018) for intravenous Levofloxacin and increased from 6.9 ± 1.1 to 8.6 ± 1.2 (*P*-value = 0.006) for oral Levofloxacin. Increasing rate of oral Levofloxacin confirms that our recommendation for changing IV antibiotics to oral form was effective.

One of the most important limitations for implementing a comprehensive ASP in developing countries, as seen in our study, is limitation in resources (20). In the present study the clinical pharmacy specialist visited the patients in internal medicine ward with 46 beds, three times a week. Definitely, attendance of the clinical pharmacist on daily bases could have higher impact on the reduction of the antimicrobial use especially with increasing chance of communication with physicians and identifying discrepancies from the approved guidelines.

Other limitation for implementing an ASP is finding the right way to assess treatment appropriateness including optimal drug choose and treatment duration which is still under debate by specialist (11, 21). Another limitation is lack of standard definitions for defining appropriateness of treatment or effect of intervention. For example, “time to treatment modification (time to de-escalation, changing route of administration and antibiotic discontinuation) has been chosen in some studies (13, 17, 22 and 23) as the primary outcome to assess treatment appropriateness. Although this outcome is easier to assess, but it is not necessarily equivalent to an improvement in the appropriateness (17). Additionally, we did not assess program influence on bacterial resistance. For evaluating these important issue larger studies, implementing such strategies in whole hospital with longer duration, is recommended.

In conclusion, the current study revealed that post prescription review of IV antibiotics and feedback is an effective intervention to increase appropriateness of the treatment and to reduce the broad-spectrum antibiotic usage. This research has also demonstrated that this intervention could decrease the hospital length of stay with no effect on mortality.
